# Diabetic peripheral neuropathy among adult type 2 diabetes patients in Adama, Ethiopia: health facility-based study

**DOI:** 10.1038/s41598-024-53951-y

**Published:** 2024-02-15

**Authors:** Yohannes Mekuria Negussie, Nardos Tilahun Bekele

**Affiliations:** 1 Department of Medicine, Adama General Hospital and Medical College, Adama, Ethiopia; 2 Department of Public Health, Adama Hospital Medical College, Adama, Ethiopia

**Keywords:** Diabetic neuropathy, Diabetes mellitus, Adama, Ethiopia, Diabetes complications, Type 2 diabetes, Epidemiology

## Abstract

Diabetic peripheral neuropathy is the most prominent microvascular complication of diabetes mellitus and the leading cause of ulceration, amputation, and extended hospitalization. Evidence regarding the magnitude and factors associated with diabetic peripheral neuropathy is not well documented in Ethiopia, particularly in the study area. A facility-based cross-sectional study was conducted among 293 adult type 2 diabetic patients who were on treatment and follow-up from May to June 31, 2023. To select participants in the study, a systematic random sampling method was utilized. Data were collected using semi-structured questionnaires and medical record reviews. The Michigan Neuropathy Screening Instrument (MNSI) was employed to assess diabetic peripheral neuropathy. To model the association between diabetic peripheral neuropathy and independent variables, binary logistic regression model was used. An adjusted odds ratio with a 95% confidence interval was used to estimate the association and statistical significance was proclaimed at a p-value < 0.05. The magnitude of diabetic peripheral neuropathy was 14.3% (95% CI 10.4–18.0). It was 13.4% (95% CI 8.4–19.1) among males and 15.4% (95% CI 10.1–22.2) among females. Age above 60 years (AOR = 5.06, 95% CI 1.60–15.96), being rural resident (AOR = 2.41; 95% CI 1.15–5.06), duration of diabetes above 5 years (AOR = 2.48, 95% CI 1.16–5.27) and having comorbid hypertension (AOR = 2.56, 95% CI 1.24–5.28) were independently associated with diabetic peripheral neuropathy. One in seven adult type 2 diabetes patients in the study area had diabetic peripheral neuropathy. Factors such as age, place of residence, duration of diabetes, and comorbid hypertension showed positive associations with diabetic peripheral neuropathy. Thus, it is imperative to give special consideration to diabetic patients who are elderly, living in rural areas, experiencing a prolonged duration of diabetes, or dealing with comorbid hypertension.

## Introduction

Diabetes mellitus (DM) remains a serious public health problem, and type 2 DM is progressing rapidly, impacting individuals in all regions of the world. Every year, diabetes is directly responsible for 1.5 million deaths^1^. In 2021, it was estimated that 537 million adults globally had DM; this figure is expected to climb to 643 million by 2030 and 783 million by 2045, with low- and middle-income nations bearing the brunt of the predicted growth. Elevated mortality and morbidity rates, affecting both developing and developed nations, primarily stem from DM, with type 2 DM playing a leading role in this health burden. Ethiopia is one of the top five African countries pertaining to DM, with an estimated 1.9 million people suffering from it^1–4^.

Diabetes complications are expected to rise in tandem with the rise in the incidence of DM^5^. Blindness, renal failure, and peripheral neuropathy are all mainly triggered by DM. A quarter of individuals diagnosed with type 2 DM exhibit signs of DM complications right from the outset. Diabetic peripheral neuropathy (DPN) is the most prominent microvascular complication of DM and the most common cause of foot ulcers and amputations^6,7^. Nearly 30–50% of people with DM develop DPN throughout their lifetime and it accounts for 80% of foot ulcers, 50–75% of non-traumatic amputations, and frequent hospitalization^8–11^.

DPN is a global health issue that affects both developed and developing nations. Globally, the prevalence of DPN ranges from 22 to 47%^12–14^, while in Africa it is estimated to be 22%–66%^15–17^. In Ethiopia, the prevalence ranges from 1.9 to 53.6%, with variations contingent on geographical region and time^7,18^. Owing to late diagnosis, a paucity of screening and diagnostic resources, and a lack of proper DM care services, the prevalence of DPN is high in developing countries^19,20^.

Earlier studies have demonstrated that the development of DPN in type 2 DM patients is influenced by several factors, including age, the duration of diabetes, glycemic control, fasting blood sugar (FBS) level, body mass index (BMI), hypertension, diabetic retinopathy, and smoking^7,21–26^.

To reduce the detrimental consequences of DPN complications, it is crucial to screen type 2 DM patients for DPN at the earliest possible time. It is suggested that each adult with DM be checked for DPN at the time of diagnosis and then once a year after that. Preventing the complications and morbidity associated with DPN requires early recognition, detection, and management of risk factors^27,28^. Nevertheless, there is insufficient evidence, particularly within the study area, to demonstrate the magnitude and factors associated with DPN. Recognizing the escalating global burden of DM and its complications, our study aims to fill a critical gap in the current understanding of the magnitude and contributing factors of DPN specific to the study area. By shedding light on the nuanced dynamics of DPN in the local context, our findings are poised to play a pivotal role in informing targeted interventions, enhancing patient care, and ultimately contributing to the broader discourse on diabetes management. Therefore, this study aimed to determine the magnitude of DPN and associated factors among adult type 2 DM patients in Adama, central Ethiopia.

## Methods and materials

### Study design, setting, and period

A facility-based cross-sectional study was conducted in Adama city from May 01 to June 30, 2023. Adama city is 99 km to the southeast of Addis Ababa and is situated in one of the special zones of the Oromia region (East Shoa). The city covers 29.86 km^2^, with a population exceeding 500,000. It features one public hospital, five private hospitals, 160 private clinics, and eight health centers. They collectively provide services to catchment areas with over five million people and act as a referral hub for zones and regions nearby^29^.

### Population and eligibility criteria

The source population was all adult type 2 DM patients on follow-up at Public health facilities in Adama city. The study population was all DM patients who were on follow-up at randomly selected Public health facilities in Adama city during the study period. Patients under the age of 18 years, who were critically and mentally ill, pregnant women, and patients diagnosed during the study period were excluded.

### Sample size determination

The sample size for this study was determined using the single population proportion formula considering the assumptions of a 95% confidence level (the critical value Zα/2 = 1.96), a 5% margin of error (d = 0.05), and a 22% (p = 0.22) proportion of DPN^18^.

$$n = \frac{{\left( {z\alpha /2} \right)^{2} p\left( {1 - p} \right)}}{{d^{2} }} = 264$$

where: **n** = the required sample size, **Z α/2** = the standardized normal distribution curve value for the 95% confidence interval, **p** = the proportion of DPN, and **d** = degree of precision.

By adding 10% for nonresponse, the final sample size of the study becomes **293**.

### Sampling procedure and technique

**Figure 1:**
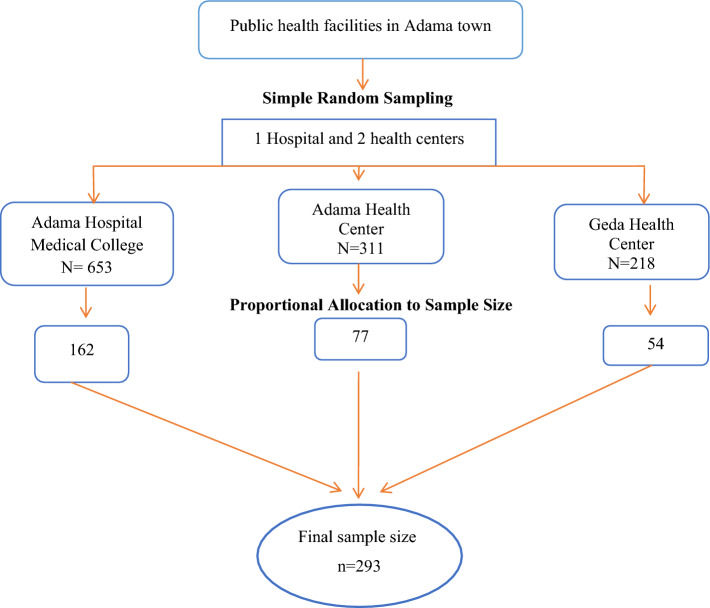
Schematic presentation of the sampling technique used to select the study participants from public health facilities of Adama, Ethiopia, 2023.

Public health facilities were stratified into hospitals and health centers. From eight health centers, two were selected by a simple random sampling method, and the hospital was selected purposively. Samples were proportionally allocated to the chosen health facilities based on their previous three-month average patient flow for DM follow-up. To recruit study participants from each facility, a systematic random sampling method was used. The sampling interval was set at each facility by dividing the average number of DM patients in the previous three months at the chosen facilities by the required sample size (Fig. 1).

### Operational definitions and study measurements

Diabetic peripheral neuropathy**:** the presence of DPN was assessed using the Michigan neuropathy screening instrument (MNSI). DPN was deemed to be present if the patient's history version of MNSI questionnaire score was ≥ 7 and/or if the examination version of MNSI scores was ≥ 2.5^7,30,31^.

The tool consists of two complementary parts. The first part is the history version. It encompasses 15 items, of which only 13 were used to assess symptoms of DPN. The total score ranges from 0 to 13 points, and a score of ≥ 7 indicates the presence of DPN.

The second part contains a physical examination of the feet. It involves inspection of the feet appearance, vibration perception at the distal great toe using a 128-Hz C-tuning fork, examination of the ankle reflex using a standard triangular rubber-headed hammer, and a 5.07/10-g Semmes–Weinstein monofilament test.

On appearance, a foot with any abnormality received a score of one, and if not 0, a foot with an ulcer received a score of 1, and if not 0, absent vibration perception received a score of 1, reduced to 0.5, and if the vibration is present, 0. If ankle reflex was present, it was scored as 0, while if absent, the patient was told to do the Jendrassic maneuver, and if present, scored 0.5 and otherwise scored 1. On the monofilament test, nine sites on the plantar surface and one on the dorsum of the foot were examined. A score of 8 correct responses out of 10 applications received 0, 1–7 correct responses received 0.5, and no correct answers received 1. After each foot was evaluated independently, the results were combined. If the patient's score on the examination version of the MNSI was ≥ 2.5 on a scale of 10, they were deemed to have DPN.

Physical activity: Participants were deemed physically active if they engaged in physical exercise for at least 30 min per day, five days per week (≥ 150 min per week)^7,32^.

Smoking: participant who has never smoked, or who has smoked less than 100 cigarettes in his or her lifetime was considered non-smoker.

Alcohol intake: Male participants who drank more than 2 units of alcohol per day and female participants who drank more than 1 unit of alcohol per day were deemed alcohol consumers.

### Data collection procedure and quality control

Data were collected using interviewer-administered semi-structured questionnaires and patient medical record reviews. The questionnaire was derived from validated scales and published studies and tailored to the study’s context^7,8,19,21,22,24,26,30^. A data extraction checklist was utilized to retrieve the necessary clinical related information from patients' records.

The questionnaire was prepared in English, then translated into local languages and back into English to ensure consistency. A pre-test was conducted on 5% of the total sample of patients (n = 15) at non-selected public health institutions to assess the quality and compatibility of the data abstraction format and questionnaire with the study's objective, and a correction was made accordingly. In addition, during data collection, Supervisors evaluated each questionnaire for completeness and consistency, and the principal investigator double-checked it daily and all collected data were cross-checked during data entry to clarify any missing data.

### Data processing and analysis

Data were exported to the Statistical Package for Social Sciences (SPSS) Version 27 for cleaning and analysis after being coded and entered into Epi-Info Vision 7. To describe the study population in the context of pertinent characteristics, descriptive statistics were used. The association between the independent and outcome variables was modeled using binary logistic regression. The model's statistical assumptions were checked, and no major violations were seen. In the bivariable logistic regression model, a p-value of 0.25 was chosen as a cut-off value to select variables for multivariable logistic regression analysis to control the effects of confounders. The multicollinearity between the explanatory variables was explored using the variance inflation factor and tolerance, and it was determined to be within the range (1.013–1.028) showing no existence of multicollinearity. The adjusted odds ratio (AOR) with a 95% confidence interval (CI) was utilized to identify factors independently associated with DPN in the multivariable logistic regression. The model was fitted using the standard model-building method. Hosmer and Lemeshow's goodness-of-fit test was used to assess the model's fitness, and the result was significant with a p-value of 0.471 indicating a good fit. In the final model, variables with a p-value less than 0.05 were considered statistically significant.

### Ethical approval and consent to participate

Ethical approval was obtained from the Institutional Ethical Review Board of Adama Hospital Medical College, and permission to access patient records and conduct the study was obtained from the medical directors of each selected health facility. Participants were informed of the study's purpose and benefits during the data collection period and informed written consent was obtained to ensure their decision to participate or refuse. To uphold respondents' rights and ensure confidentiality, anonymity, and privacy, safeguards were enacted, and all of the study's procedures followed the principles outlined in the Helsinki Declaration^33^.

## Results

### Socio-demographic characteristics

A total of 293 type 2 DM patients participated in this study giving a response rate of 100%. The mean age of the participants was 51.1 (SD: ± 12.2) years. Of the total participants, 157 (53.6%) were males, 274 (73.0%) urban residents, 223 (76.1%) married, and 119 (40.6%) were employed (Table 1).

**Table 1:** Sociodemographic characteristics of type 2 diabetes mellitus patients on follow-up at public health facilities in Adama, Ethiopia, 2023 (n = 293).

Variables	Category	Frequency	Percentage
Sex	Male	157	53.6
	Female	136	46.4
Age	≤ 40	69	23.5
	41–60	156	53.2
	> 60	68	23.2
	Mean 51.1 (SD: ± 12.2)		
Place of residence	Urban	274	73.0
	Rural	79	27.0
Marital status	Currently married	223	76.1
	Currently unmarried	70	22.8
Religion	Orthodox	148	50.5
	Muslim	106	36.2
	Protestant	39	13.3
Educational status	No formal education	27	9.2
	Elementary (1–8)	62	21.2
	Secondary (9–12)	87	29.7
	College and above	117	39.9
Occupational status	Employed	119	40.6
	Merchant	64	21.8
	Farmer	39	13.3
	Retired	47	16.1
	Others^a^	24	8.2

^a^Housewife, student, daily laborer.

### Behavioral and clinical characteristics

**Figure 2:**
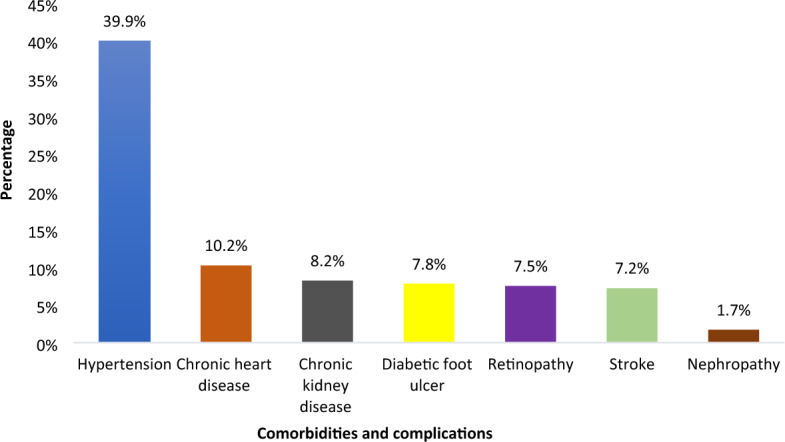
Comorbidity and complication-related characteristics of type 2 diabetes mellitus patients on follow-up at public health facilities in Adama, Ethiopia,2023 (n = 293).

Among the participants included in this study, 151 (51.5%) were taking oral hypoglycemic agents (OHAs) and the majority of DM patients, 212 (72.4%), had good glycemic control. The median FBS level was 220 (IQR = 190–292) while the median duration of DM was 3.8 (IQR = 1.8–6.1), and of the participants, 216 (73.2%) were physically inactive and 116 (39.6%) had a history of missed follow-up (Table 2). In this study, hypertension 117 (39.9%) was the most common comorbidity, followed by chronic heart disease (CHD) 30 (10.2%) and chronic kidney disease 24 (8.2%). The commonest complication was diabetic foot ulcer 23 (7.8%) (Fig. 2).

**Table 2:** Behavioral and clinical characteristics of type 2 diabetes mellitus patients on follow-up at public health facilities in Adama, Ethiopia, 2023 (n = 293).

Variables	Category	Frequency	Percentage
Type of treatment	Oral hypoglycemic agents	151	51.5
	Insulin	37	12.6
	Mixed	105	35.8
Oral hypoglycemic agents	Metformin	133	45.4
	Glibenclimide	45	15.4
	Both	115	39.2
Fasting blood sugar level (mg/dl)	< 200	101	34.5
	≥ 200	192	65.5
	Median 220 (IQR = 190–292)		
Glycemic control	Good	212	72.4
	Poor	81	27.6
Body mass index (kg/m^2^)	Underweight	32	10.9
	Normal (18.5–24.99)	116	39.6
	Overweight (25–29.99)	78	26.6
	Obese (≥ 30)	67	22.9
Family history of DM	Yes	21	8.2
	No	272	92.8
Duration of DM	< 5 years	223	76.1
	≥ 5 years	70	23.9
	Median 3.8 (IQR = 1.8–6.1)		
Physical activity	Active	77	26.3
	Inactive	216	73.2
Alcohol intake	Yes	36	12.3
	No	257	87.7
History of Smoking	Yes	9	3.1
	No	284	96.9
Follow-up miss	Yes	116	39.6
	No	177	60.4

### The magnitude of diabetic peripheral neuropathy

**Figure 3:**
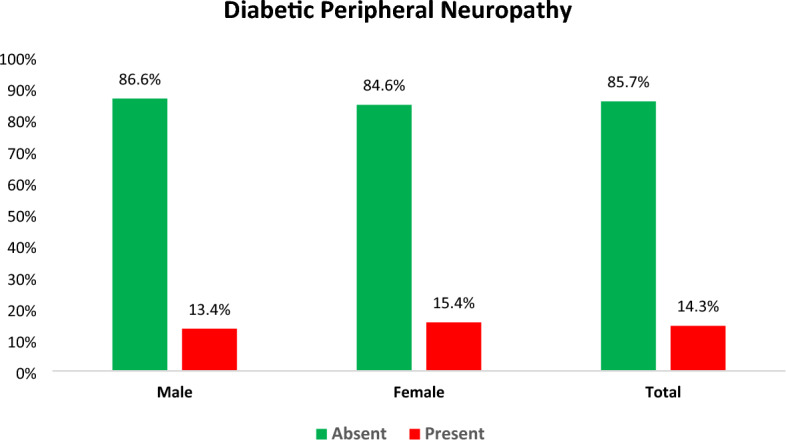
The proportion of diabetic peripheral neuropathy among type 2 diabetes mellitus patients on follow-up at public health facilities in Adama, Ethiopia, 2023 (n = 293).

The proportion of DPN among type 2 DM participants was 14.3% with (95% CI 10.4–18.0). It was found to be 13.4% (95% CI 8.4–19.1) among males and 15.4% (95% CI 10.1–22.2) among females respectively (Fig. 3).

### Factors associated with diabetic peripheral neuropathy

In bivariable analysis, age, place of residence, type of treatment, glycemic control duration of DM, and comorbid HTN demonstrated a significant association. After adjusting for potential confounders using multivariable binary logistic regression analysis age, place of residence, duration of DM, and comorbid HTN persisted as statistically significant factors associated with DPN at a p-value < 0.05.

The odds of developing DPN were 5 times greater among DM patients above the age of 60 compared to patients 40 years of age or younger (AOR = 5.06, 95% CI 1.60–15.96). Patients who resided in rural areas had 2.4 times the odds of experiencing DPN than those living in urban areas (AOR = 2.41; 95% CI 1.15–5.06). Compared to patients with DM for 5 years or less, those with DM for more than 5 years had 2.4 times the odds of developing DPN (AOR = 2.48, 95% CI 1.16–5.27). Furthermore, the odds of developing DPN among DM patients with comorbid HTN were 2.5 times higher compared to their counterparts (AOR = 2.56, 95% CI 1.24–5.28) (Table 3).

**Table 3:** Factors associated with diabetic peripheral neuropathy among type 2 diabetes mellitus patients on follow-up at public health facilities in Adama, Ethiopia, 2023 (n = 293).

Variables	Category	DPN		COR (95% CI)	AOR (95% CI)
		Present (%)	Absent (%)		
Age (years)	≤ 40	5 (7.2)	64 (92.8)	1	1
	41–60	20 (12.8)	136 (87.2)	1.88 (0.68–5.24)*	1.77 (0.60–5.23)
	> 60	17 (25.0)	51 (75.0)	4.27 (1.47–12.35)*	5.06 (1.60–15.96)**
Residence	Urban	25 (11.7)	189 (88.3)	1	1
	Rural	17 (21.5)	62 (78.5)	2.07 (1.05–4.09)*	2.41 (1.15–5.06)**
Type of treatment	OHA	24 (15.9)	127 (84.1)	1	1
	Insulin	3 (8.1)	34 (91.9)	0.47 (0.13–1.64)*	0.53 (0.14–2.05)
	Mixed	15 (14.3)	90 (85.7)	0.88 (0.44–1.78)	0.81 (0.38–1.72)
Glycemic control	Good	25 (11.8)	187 (88.2)	0.50 (0.26–0.99)*	0.46 (0.22–1.01)
	Poor	17 (21.0)	64 (79.0)	1	1
Duration of DM (years)	≤ 5	26 (11.7)	197(88.3)	1	1
	> 5	16 (22.9)	54(77.1)	2.25(1.12–4.84)*	2.48(1.16–5.27)**
Comorbid HTN	Yes	24 (20.8)	93 (79.5)	2.27 (1.17–4.39)*	2.56 (1.24–5.28)**
	No	18 (10.2)	158(89.8)	1	1

## Discussion

This health facility-based cross-sectional study assessed the magnitude and factors associated with DPN among adult type 2 DM patients in Adama, Ethiopia.

This study revealed that the magnitude of DPN was 14.3% (95% CI 10.4–18.0) which is comparable with studies conducted in Spain (12.9%), Ghana (16.6%)^34^, and Northwest Ethiopia (16.63%)^24^. Conversely, the finding of the current study is lower than a global systematic review and metanalysis (35.78%)^13^, a systematic review and metanalysis done in Africa (46%)^17^, studies done in Singapore (28%)^35^**,** Vietnam (26.6%)^36^ Jimma University Medical Center, Ethiopia (53.6%)^7^, and Bahirdar, Ethiopia (52.2%)^26^. Moreover, the magnitude of DPN in this study is higher than in studies conducted in Nigeria (7.5%)^37^ and China (8.4%)^38^**.** These discrepancies could be attributed to variations in neuropathy diagnostic criteria and differences in the study population, with the present study encompassing individuals with type 2 DM. Additionally, divergences in socioeconomic status, study period, sample size, and study settings may offer another plausible explanation for the noted differences.

In line with studies conducted in China^39^, Singapore^35^, India^40^, Romania^41^, Egypt^42^, Uganda^43^, Tanzania^44^, Northwest Ethiopia^24,25^, Jimma University Medical Center, Ethiopia^7^, and Bahirdar Ethiopia^26^. The odds of developing DPN were fivefold greater among DM patients above the age of 60 compared to patients 40 years of age or younger. The justification lies in the age-related increase in oxidative stress, counter-regulatory signaling stimulation, and mitochondrial dysfunction. These factors heighten the risk of inflammation and peripheral nerve damage, consequently escalating the likelihood of DPN. Additionally, aging exposes the neural system to continual metabolic stress and declining physiological health, further elevating the risk of contracting DPN^45–47^.

Place of residence was another significant factor associated with DPN. In this study, Patients who resided in rural areas had 2.4 times the odds of experiencing DPN than those living in urban areas. This finding is consistent with previous studies conducted in Sri Lanka^20^ and India^48^. This may be due to a lack of better healthcare facilities to detect and treat diabetic problems in rural settings. On the contrary, studies done in Cameroon^19^ and Gamo and Gofa zones, Ethiopia^22^ showed higher odds of DPN among urban dwellers. This disparity may be attributable to the majority of urban people maintaining sedentary lifestyles, unhealthy eating habits, and less physical activity all of which can contribute to DPN^49–51^.

In this study, there were also statistically significant associations between the duration of DM and higher odds of DPN. So, compared to patients with DM for 5 years or less, those with DM for more than 5 years had 2.4 times the odds of developing DPN. This finding is similar to a systematic review and meta-analysis of 16 studies^23,52^; studies done in China^38^, Jordan^53^, India^54^ Egypt^55^, Iran^56^, Jimma University Medical Center, Ethiopia^7^; Bahirdar, Ethiopia^26^; Gamo and Gofa zones, Ethiopia^22^. This can be explained by the fact that the prolonged duration of DM is linked with chronic hyperglycemia, which activates several metabolic pathways, causing oxidative stress in diabetic neurons and leading to nerve damage and neuronal ischemia^57,58^. Moreover, living with diseases for an extended time may raise the likelihood of being exposed to unhealthy lifestyles and foods raising the risk of developing complications^22^. Conversely, a study conducted in Northwest Ethiopia showed a contrasting finding^24^.

The finding of this study indicated that the odds of DPN were greater among DM patients with comorbid HTN compared to their counterparts. This is supported by findings from a systematic review and meta-analysis of 16 studies^52^, a finding from a systematic review^59^, and studies conducted in China^39,60^, Taiwan^61^, Egypt^42^, and Northwest Ethiopia^25^. Increased oxidative stress, inflammation, activation of the sympathetic nervous system, stimulation of the renin–angiotensin–aldosterone system, endothelial cell dysfunction, vascular rigidity leading to reduced blood supply to the nerve cell, slowed conduction, and axonal atrophy—each of these factors may contribute to the increased susceptibility to developing DPN as a result of HTN^62,63^. The mechanism of HTN in DPN is still obscure, and further studies are required to define the nature of the physiologic interaction, as well as the relationship between HTN and DPN in diabetics.

### Limitations of the study

Given the cross-sectional nature of the study, it is difficult to draw a causal relationship, and the gold standard diagnostic test for DPN was not used due to its high cost.

## Conclusion

This study revealed that one in seven DM patients had DPN. Age, place of residence, duration of DM, and comorbid HTN were factors positively associated with DPN. Therefore, it is essential to prioritize diabetic patients, including the elderly, those in rural areas, individuals with a longer duration of diabetes, and those with comorbid hypertension, during routine follow-up visits. Tailored care and focused monitoring for these groups are crucial for effective healthcare management, thereby enhancing diabetes management effectiveness.

## Data Availability

All data and materials are available from the corresponding author without undue reservation.

## Abbreviations

AHMC: Adama Hospital Medical College
AHR: Adjusted hazard ratio
CI: Confidence interval
DM: Diabetes mellitus
DPN: Diabetic peripheral neuropathy
FBS: Fasting blood sugar
HTN: Hypertension
MNSI: Michigan neuropathy screening instrument
OHA: Oral hypoglycemic agent
